# How are Research for Development Programmes Implementing and Evaluating Equitable Partnerships to Address Power Asymmetries?

**DOI:** 10.1057/s41287-023-00578-w

**Published:** 2023-02-23

**Authors:** Mieke Snijder, Rosie Steege, Michelle Callander, Michel Wahome, M. Feisal Rahman, Marina Apgar, Sally Theobald, Louise J. Bracken, Laura Dean, Bintu Mansaray, Prasanna Saligram, Surekha Garimella, Sophia Arthurs-Hartnett, Robinson Karuga, Adriana Elizabeth Mejía Artieda, Victoria Chengo, Joanes Ateles

**Affiliations:** 1 Tomorrow’s Cities Urban Disaster Risk Hub, Institute of Development Studies, Falmer, Brighton, UK; 2 Tomorrow’s Cities Urban Disaster Risk Hub, Facultad Latinoamericana de Ciencias Sociales Sede (FLACSO), Quito, Ecuador; 3 Tomorrow’s Cities Urban Disaster Risk Hub, African Centre for Technology Studies (ACTs), Nairobi, Kenya; 4 ARISE Hub, Liverpool School of Tropical Medicine, Liverpool, UK; 5 ARISE Hub, George Institute for Global Health India, New Delhi, India; 6 ARISE Hub, College of Medicine and Allied Health Sciences, Freetown, Sierra Leone; 7 ARISE Hub, LVCT Health, Nairobi, Kenya; 8 Gender and Justice Security Hub, London School of Economics and Political Science, London, UK; 9 Living Deltas Hub, Northumbria University, Newcastle Upon Tyne, UK; 10 One Ocean Hub, University of Strathclyde, Glasgow, UK

**Keywords:** Research for development, Equitable partnerships, Evaluation research, Theory-based evaluation, Participatory evaluation, Monitoring, evaluation and learning

## Abstract

The complexity of issues addressed by research for development (R4D) requires collaborations between partners from a range of disciplines and cultural contexts. Power asymmetries within such partnerships may obstruct the fair distribution of resources, responsibilities and benefits across all partners. This paper presents a cross-case analysis of five R4D partnership evaluations, their methods and how they unearthed and addressed power asymmetries. It contributes to the field of R4D partnership evaluations by detailing approaches and methods employed to evaluate these partnerships. Theory-based evaluations deepened understandings of how equitable partnerships contribute to R4D generating impact and centring the relational side of R4D. Participatory approaches that involved all partners in developing and evaluating partnership principles ensured contextually appropriate definitions and a focus on what partners value.

**Supplementary Information:**

The online version contains supplementary material available at 10.1057/s41287-023-00578-w.

## Résumé

Finally, centring reflexivity within a learning oriented approach ensured that partnership evaluation findings were used to adapt and improve the way R4D programmes operate. La complexité des enjeux abordés par la recherche pour le développement (R4D) nécessite des collaborations entre des partenaires de disciplines et de contextes culturels variés. Les asymétries de pouvoir au sein d'un tel partenariat peuvent entraver la répartition équitable des ressources, des responsabilités et des avantages entre tous les partenaires. Cet article présente une analyse croisée de cinq évaluations de partenariats R4D, leurs méthodes et la manière dont elles ont mis au jour et traité les asymétries de pouvoir. Cet article contribue au domaine des évaluations de partenariats R4D en décrivant de façon détaillée les approches et les méthodes employées pour évaluer ces partenariats. Les évaluations basées sur la théorie ont permis d'approfondir la compréhension de la manière dont les partenariats équitables contribuent à l'impact de la R4D et à mettre au centre l'aspect relationnel de la R4D. Les approches participatives impliquant tous les partenaires dans l'élaboration et l'évaluation des principes du partenariat ont permis de garantir des définitions adaptées au contexte et de faire en sorte que les évaluations se penchent sur ce qui est jugé important pour tous les partenaires. Enfin, le fait de mettre la réflexivité au cœur de l’analyse dans une approche axée sur l'apprentissage a permis d'utiliser les résultats de l'évaluation du partenariat pour adapter et améliorer le fonctionnement des programmes de R4D.

## Introduction

The complexity of the issues addressed by research for development (R4D) programmes requires collaborations between partners from a wide range of backgrounds, disciplines and contexts. Indeed, research partnerships with development actors have been identified as crucial to support achievement of the Sustainable Development Goals (SGDs) (DFID, 2016) and achieve lasting impact (Georgalakis and Rose 2019).

Funders of R4D are actively prioritising equitable partnerships to ensure resources, responsibilities and outcomes of the programmes they fund are shared fairly across partners and that inputs from all partners are optimally incorporated (Barr et al. 2018; Fransman et al. 2021). Implementing the concept of *equity* in practice, however, remains difficult in part due to power asymmetries that characterise the dominant colonial, Eurocentric and patriarchal context in which R4D operates. Power asymmetries can be conceptualised through different lenses, for example through geographical/colonial, gender, and academic hierarchies that can take intersecting forms. Colonial legacies shape the flow of R4D funding from Higher Income Country (HIC) governments or philanthropic organisations, which often mandate the lead institution to be based in HIC. For example, the Global Challenges Research Fund which is funded through UK Overseas Development Assistance [and administered by UK Research and Innovation (UKRI)] requires principal investigators to be based at a UK Higher Education Institution. Further, they require lead institutions to undertake stringent due-diligence processes on partners they sub-contract in Low and Middle Income Countries (LMIC), arguably setting the tone for unequal decision-making and division of labour from the outset (Price et al. 2021). It is well recognised that academia in general prioritises certain forms of knowledge production over others (Smith 2012). In the context of R4D, this can translate into prioritising research for the production of high-quality academic outputs over the action-oriented focus of research undertaken by applied research partners such as NGOs or a focus on technical research tasks over the relational side of R4D partnership working (Thomas Archibald 2020). A related dynamic is the marked hierarchy between senior academics who tend to be based in the UK, and early career researchers (ECRs) who are more often based in LMIC. Gendered power imbalances further influence degrees of equity in partnerships in R4D, with cisgendered male academics and partners often having more opportunities and access to resources than those of other genders.

Paying close attention to how R4D partnerships take shape, and evaluating equity within and through them could, therefore, generate knowledge and learning to help address hard to shift power asymmetries. Further, sharing evaluation findings across complex, international, inter-organisational partnerships can contribute to improving R4D practice and, consequently, achieving greater impact. There are limited partnership evaluations in the context of R4D programming, and those that exist are based on single cases, lacking descriptions of their evaluation approaches which limits their potential use (Price et al. 2021). We respond to this gap by (1) detailing the approaches and methods employed to evaluate equitable partnerships in five large R4D programmes and, (2) presenting cross-case analysis on how power asymmetries were unearthed (what the evaluations found) and addressed (how the findings were used).

We, the authors, represent different voices and perspectives within the five R4D cases presented in the paper. We are early career (ECR) and senior researchers, programme managers and monitoring, evaluation and learning (MEL) professionals from UK-based and LMIC-based institutions and/or backgrounds. This paper resulted from a series of online and email discussions held over 2020 to 2022 to share and reflect on evaluation across the R4D programmes. We identified that equitability in our partnerships was mainly challenged by three power asymmetries driven by: (i) geographical location of partners, (ii) academic/knowledge hierarchies and (iii) gender relations. As with any analytical frame, these present a simplification of reality and there are a myriad of other intersecting power dynamics at play between partners, but these covered the most salient dynamics that were surfaced through the evaluations and our reflections on them. We use a multiple case study approach (Marrelli 2007), within which each R4D programme is a unique case using unique evaluation methods. We compared across the cases to identify similarities and differences in our evaluation approaches and use of findings. This paper begins with an overview of the five cases, including their approaches to partnership working (“Case Descriptions: Overview of Approaches to Equitable Partnerships” section), followed by a description of the evaluation methods that were used in each (“Case Evaluation Methods” section). The results of these evaluations and how the results were used to address power asymmetries are synthesised across the five cases and presented in “Cross-case Analysis Findings” section. We end with a discussion on implications for equitable partnership evaluations in R4D.

## Case Descriptions: Overview of Approaches to Equitable Partnerships

The included cases were five R4D programmes (hereafter referred to as “the Hubs”) funded in 2019 by the Global Challenge Research Fund (GCRF) as 5-year, interdisciplinary Hubs to address intractable challenges (UKRI 2019). The Hubs conduct world-class research to improve outcomes for marginalised people in LMICs by working with a large number of academic and non-academic partners in a wide variety of geographies in LMICs (Table 1). At the time of writing, the Hubs are in their third year and have faced multiple crises, including: the Covid-19 pandemic; unexpected budget cuts of up to 70% as part of the UK government’s reduction in foreign aid spending in 2021 (Brien and Loft 2022; Nwako et al. 2023); various national environmental and socio-political crises within partner countries (including the Taliban regime taking over in Afghanistan in 2022 and flooding in Sierra Leone in 2019 and 2022). These multiple crises led to interruptions to field work and consequently to partnership working.

**Table 1:** Challenge-driven research for development Hubs

Name	Thematic focus	Geographical focus	No. and type of partners	Amount awarded
ARISE	This Hub supports people who have been marginalised to claim their health rights and helps build government accountability and capacity through participatory research and co-production approaches with communities and governance actors to inform policy change at all levels	Bangladesh, India, Kenya, Sierra Leone	11 (4 HIC, 7 LMIC; 6 universities, 4 LMIC research organisations, 1 NGO/CSO)	£12.1 M
Gender, Justice and Security hub (GJS)	The Gender, Justice and Security Hub is a multi-partner research network working with local and global civil society, practitioners, governments and international organisations to advance gender justice and inclusive peace	Afghanistan, Colombia, Iraq, Lebanon, Sierra Leone, Sri Lanka, Uganda	40 (16 HIC; 24 LMIC; 24 universities, 4 LMIC research organisations, 14 NGO/CSO)	£15.2 M
Living Deltas (LD)	Focusing on three deltas in Asia, this Hub operates on a model of equitable partnership with the delta-dwellers and the research community working together to develop new knowledge and policies. The aim is to safeguard delta futures through more resilient communities and sustainable development	Bangladesh, India and Vietnam	22 (11 HIC-UMIC/11 LMIC; 16 Universities; 5 research institutes; 1 NGO)	£15.3 M
One Ocean Hub (OOH)	This Hub bridges current connects across law, science and policy to empower to co-develop research and solutions to challenges facing ocean ecosystems and coastal communities, and transform ocean governance	Ghana, Namibia, South Africa, Fiji and Solomon Islands	78 (HEIs, government research organisations, NGO, and CSOs)	£18.2 M
Tomorrow’s Cities (TC)	This Hub works with international agencies to bring disaster risk management to the centre of global urban policy and practice, strengthening the voice and capacity of the urban poor	Ecuador, Nepal, Kenya and Turkey	54 (29 HIC / 25 LMIC; 23 universities; 6 CSOs; 9 NGOs; 2 government; 3 private sector; 12 research institute)	£17.6 M

These numbers and details on partners and budget are the original numbers, before the budgets were cut by 70% in 2021

The GCRF identified equitable partnerships as one of the key outcome areas in its theory of change (Barr et al. 2018). While no explicit definition of equitability is provided by GCRF, UKRI provides guidance to grantees that equitable partnerships should be characterised by joint ownership, mutual responsibility, transparency and benefits for all partners. This characterisation was built on and therefore aligns with existing literature (Price et al. 2021). All five Hubs are implementing a decolonial, feminist and/or participatory approach in order to actively strengthen the equitability of their partnerships (Table 2). We found considerable overlap between these approaches, in particular around how they are explicitly addressing power, decentring positivist epistemologies and including indigenous knowledge. As a simplification, the main difference between the approaches is the key power asymmetry that is the starting point of each: (i) decolonial approaches challenge historical colonial power dynamics; (ii) feminist approaches challenging patriarchal power dynamics and; (iii)participatory approaches are about all forms of power that can lead to exclusion and aims to centre those who are excluded.

**Table 2:** Principles and elements of different partnership approaches

Decolonised approaches	Feminist approaches	Participatory approaches
Principles ARISE Hub: 1. Equity in voice, power and resource distribution; 2. Transparency and accountability in priority-setting, decision-making, data and resource use; 3. Continuous co-learning, based on respectful relationships, flexibility and reflexive practice; 4. A commitment to ethical interactions at all levels of the programme	1. Be attentive to, and work towards overcoming, power inequalities and inequitable gender dynamics 2. Build equitable partnerships 3. Adhere to the highest ethical standards in our research design and practice, knowledge production, knowledge dissemination, impact and capacity building work 4. Seek to ensure equitable access to resources, opportunities and prestige 5. Develop cooperative, collaborative, inclusive and, as far as possible, transparent decision-making processes, 6. Positive and respectful communication that supports inclusion of marginalised voices and diversity of views 7. Support healthy work-life balance, career development and capacity building 8. Engage in open and honest discussion about concerns or difficulties as soon as possible after they arise 9. Practice regular critical self-reflection and remain open and accountable to feedback	Partnership elements Tomorrow’s Cities: • Respect • power relations, • having a shared mission, • clear roles and responsibilities, • communication, • participation, • transparency • fair distribution, • mutuality and co-ownership, • learning and accountability

*Decolonial approaches* to equitable partnerships aim to address historic colonial power relations that are perpetuated by existing research governance and funding structures. In the cases of the ARISE, Living Deltas (LD), Gender Justice and Security (GJS) and OneOcean (OOH) Hubs this intent is operationalised through prioritising transdisciplinarity (the integration of methods and knowledges from different disciplines and stakeholders outside of academia developed with local stakeholders to work on locally identified challenges) and employing a decolonial ethics of care and decolonial approach to safeguarding. The ARISE hub have documented their approach, highlighting reflective practice and critical thinking about power, judgement and positionality and the ownership of the global narrative surrounding safeguarding (Aktar et al. 2020; Mansaray et al. 2022). Examples of specific practices of transdisciplinary in the cases include combining arts, humanities, natural and social sciences in one team that moves away from prioritising one over the other (LD) or having local partners leading discussions on how to shape the work of the Hub (OOH). ARISE has centred the knowledge of non-academic partners through the community based participatory action research (CBPR) approach, which features in the programme’s theory of change (Tremblay et al. 2018). CBPR challenges power relationships that are inherently embedded in western knowledge production by creating respectful relationships and sharing power between the researcher and the researched, prioritising equity at all levels of research partnerships.

A decolonised *ethics of care* aims to provide equitable opportunities between research actors, irrespective of gender, age or career stage, focusing on, for example, the prioritisation of PhD opportunities for LMIC partners (ARISE has 7 offsite PhD students from Bangladesh, Kenya, India and Sierra Leone whose PhD fees are covered by the lead institution’s contribution to the project). Equity in opportunity for authorship is established in the authorship guidelines, and is another practical application of the approach. The ethics of care is evaluated in ARISE’s partnership survey, which informs adaptations such as: new provisions of call down support for counselling (in person or online); establishing ECR groups with representation on executive meetings (the main decision making body in the hub); and a multi-directional mentoring scheme. LD and GJS Hubs prioritise their Flexible Funding^1^ to be committed to projects that are led by LMIC partners.

Using a *feminist approach* to equitable partnerships means centring power imbalances and seeking to dismantle hierarchical relationships, promoting collaboration and co-design across partners and disciplines, and recognising and valuing the specific expertise that each partner and each sector bring. The GJS Hub’s feminist approach to ethics comprises nine principles that underpin the Hub’s approach to research, practice and relationships (Table 2). ARISE’s CBPR approach also draws upon feminist epistemologies, by seeking to transform gendered power hierarchies through emancipatory co-production approaches that raise awareness and promote social change at community level (ARISE 2022). Such approaches are then adapted to be accessible to population groups with diverse communication needs, such as people living with disability. OOH partners co-produced a Code of Practice that is informed by a feminist ethics of care. However, this was developed primarily with the academic partners. UKRI guidelines restrict funding levels of non-academic partners which in turn limits their ability to participate in important aspects of the Hub.

*Participatory approaches* were used in the cases of the GJS, ARISE and Tomorrow’s Cities (TC) Hubs to define what equitable partnerships mean, building their own contextualised approach with all partners. The Hubs then operationalise these co-produced understandings through implementing partnership agreements, ensuring equal representation of funded partners in decision making spaces and involvement in critical reflection and problem-solving. For example, in ARISE and the TC Hub, definitions of working in equitable partnerships were developed with teams from each of the countries of operation, including identifying key elements and co-creating values of partnership working. The TC Hub aimed to move away from the dominance of HIC partners in agenda setting around equitable partnerships to be driven by LMIC perspectives (Price et al. 2021). In practice this led to the development of local partnership definitions and elements within each country team, such that contextual factors were acknowledged as driving what equity means. To illustrate, the Ecuador team highlighted the human relational side of partnership by using the term “relationship”. The Nepal team focused on embracing local knowledge and expertise. The GJS Hub model of equitable partnerships also embeds participatory decision making at both a Hub and a project level. Supporting this participatory decision-making approach is a communications strategy committed to transparency, inclusion and access to information and opportunities.

## Case Evaluation Methods

The methods used in each case varied as the purpose of partnership evaluations differed across the Hubs. Building ‘equitable partnerships’ was a key GCRF outcome area that Hubs were required to contribute to. UKRI, therefore, introduced from the outset reporting requirements for each Hub to show not just if but also *how* they are implementing equitable partnerships in practice. For some cases the partnership evaluation was primarily a part of programmatic MEL to inform reporting to UKRI (performance-based monitoring). In other cases, working in equitable partnerships was a core part of the theory of change and broader impact evaluation designs, which led to use of a theory-based approach to evaluation of equitable partnerships (Table 3). We describe each of these approaches in turn.

**Table 3:** partnership evaluation methods, tools and impacts of budget cuts and covid-19

Hub	Evaluation approaches and methods	Specific tools used	Budget cuts and covid-19 impacts
ARISE	Participatory evaluation and theory of change	• Partnership survey • Qualitative interviews and discussions with partners • Evaluation and reflection sessions following partnership meetings	Moving to online format and trying to enable conducive space for critical discussion digitally
Gender, Justice and Security	Evaluation as part of programmatic MEL	• Social network analysis^a^ • Six-monthly project progress reports • Evaluations of six-monthly Conventions	The social network analysis budget was cut after one round of data collection. Hub Conventions are now online; were proposed to be hybrid, but there is no budget for in-person meetings
Living Deltas	Theory of change	• Online questionnaire survey • Interviewing key individuals • Discussion at Annual Meeting	Focus shifted towards online and desk-based MEL activities
OneOcean	Evaluation as part of programmatic MEL Theory of Change	• Theories of change and logframes • Pathways to impact • Participant observation • Interviews • Annual questionnaire • Biannual programme report	MEL data were used to determine how budget cuts would be administered. Criteria which privileged activities that were close to completion, were directly impacting non-academic stakeholders and were in-country and UK government priorities were preserved. Due to uncertainties, some key staff decided to leave the Hub
Tomorrow’s Cities	Theory-based and participatory evaluation	• Social Network Analysis^a^ • Rubric • After action reviews (AARs)	The Hub had to scale down it’s MEL activities and took out a planned surveys and ongoing AARs

^a^See Apgar et al. (this issue)

### Evaluation as Part of Programmatic MEL

In the context of large and multi-partner programmes working in complex systems, MEL is designed not simply for reporting towards funders (to demonstrate accountability) but, more importantly, as a vehicle to support adaptive programming (Hargreaves and Podems 2012; Patton 2010). Surfacing and capturing learning then becomes central to the purpose of MEL functions which are best understood as part of iterative decision making (Ramalingam et al. 2019). Monitoring focuses on tracking how change is happening real-time, in order to learn about what is or is not working, while evaluation specifically looks at if and how the programme’s activities are contributing to change outside of the programme (outcomes and impact). Monitoring and evaluation data can bring to light programmatic issues to determine the specifics around which interventions are taking place. Across the cases the partnership evaluation, as part of programmatic MEL, contributed to accountability to the funder to inform reporting on the equitable partnership outcome area determined by UKRI. It also provided learning about how the Hub partners are working together and what areas require improvement.

**Fig. 1:**
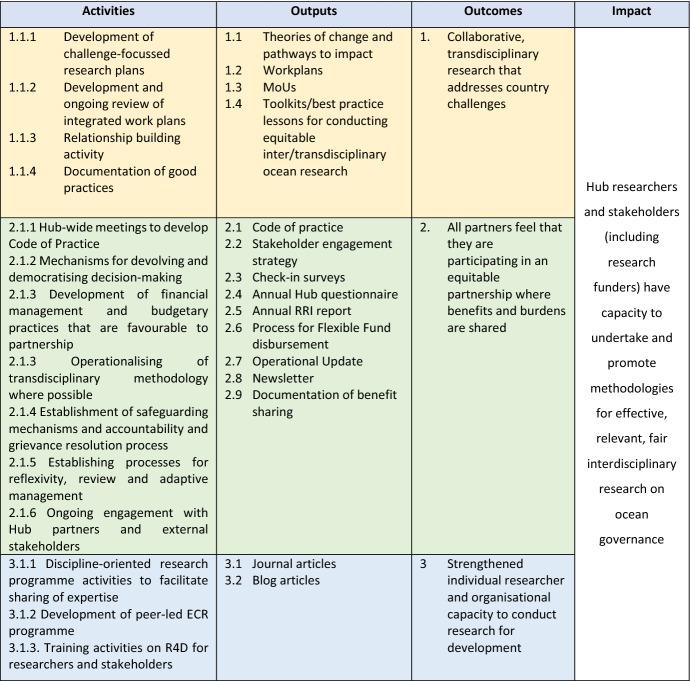
OneOcean Hub theory of change that reflects GCRF key principles

In the OOH case, the ongoing partnership evaluation provided valuable data for the hub manager to inform day-to-day functioning of the hub, as well as, over time be able to observe the Hub’s long-term trajectory. In combination, this enabled the Hub to produce evidence about adherence to the GCRF Key Principle of equitable partnerships. Operationally, the OOH Hub developed theories of change for each of its 5 countries, as well as an overarching theory of change for the whole programme. The overarching theory of change (Fig. 1) includes the activities undertaken with all partners that result in outputs that contribute to building equitable and fair partnerships. Programmatic MEL monitored progress of activities and outputs as signals that processes amenable to equitable partnerships were being operationalised and implemented. For example, how processes for reflexivity and adaptive management are implemented. In this case, ethnography conducted by a dedicated research fellow was chosen as their primary methodology as it allowed the analysis of processes as they occurred in real time. The ethnography included observations of Hub interactions and documentations combined with interviews and an annual questionnaire. The interview and questionnaire focused on Hub partners’ perception on the effectiveness of the partnership processes that were observed during interactions and in the documentation. The ethnographic research fellow conducted this research coordinated with the programme manager to make real-time changes, and also generated an annual report of their findings for the Executive Team.

The GJS Hub has developed a set of impact measures to assess, review and refine the approach to equitable partnerships. These measures appear in Hub surveys and in different parts of quarterly and annual reporting. The measures include collaborations with local partner organisations, academics and activists to influence institutional reform or preserve existing institutions and influence media representation of gender and transitional justice; the number of partner-driven publications produced by Hub members; the number of stakeholders reached through structured stakeholder analyses; and the number and quality of partner institutional development activities. The Hub then used the information gathered through the reporting and the surveys to inform their decisions to respond to the funding cuts and helped them to adapt their ways of working at the start of the pandemic.

### Theory-Based Evaluation Approaches

Theory-based evaluations are approaches that help us to understand how and why a programme, policy or intervention works, through unearthing underlying mechanisms (Rogers and Weiss 2007). It not only shows the activities, outputs and outcomes generated, but shows how and why the programme activities generate these outputs and outcomes and what context and activities need to be in place for this to happen or can obstruct this from happening (Leeuw 2003). Theory-based evaluations are particularly useful in emergent fields where there is a weak evidence base, because of their focus on trying to understand how an intervention works to generate outcomes.

**Fig. 2:**
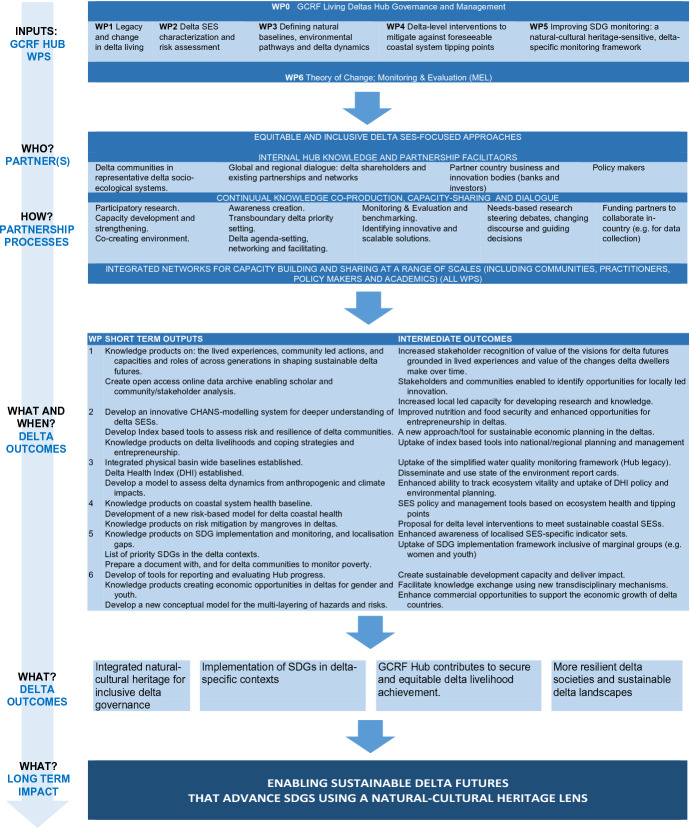
LD Hub theory of change

All the included cases had a theory of change that details how and why their research and other activities will generate outcomes and impact. Development was a funder requirement (see Chapman et al. 2023). These theories of change contain all the expected components: activities, outputs, outcomes and impacts, the causal links that join them together and the underlying assumptions and risks (Goodier et al. 2018). Given the focus of the partnership evaluations is to understand if and how working equitably does or does not enable the Hubs to address power asymmetries and generate impacts, equitable partnerships were weaved into the theories of change, yet this was done in different ways across the cases. They are included as an ongoing activity (TC, GJS and LD), as outputs (ARISE, GJS and OOH) and as outcomes (OOH and GJS) and in all cases are explicitly part of underlying assumptions about how the Hubs will achieve their outcomes. These initial theories about partnerships focus the evaluation designs on how equitable partnerships are implemented and if and then how they may contribute to outcomes. To illustrate, in the case of the LD Hub equitable partnerships are a central activity in the theory of change (Fig. 2). The equitable partnership evaluation process, then, has been tied to the MEL workplan, capturing the Hub’s existing mode of operations that directly support equity (transparency and accountability; effective management; ethical and impactful research, safeguarding and capacity strengthening). These areas were measured using an annual online survey and by interviewing the Hub’s primary investigator and manager about the current practices and experiences of these elements. Over 60% (70 responses) of the Hub members shared their responses and the data were both qualitatively and quantitatively analysed and presented before the Hub members during the Hub’s 2021 annual meeting.

### Participatory Theory-Based Evaluation Approaches

Three of the cases (GJS, ARISE and TC) used participatory theory-based evaluation approaches. Participatory evaluation approaches involve the inclusion of Hub stakeholders, paying careful attention to how marginalised stakeholders (e.g. colleagues in LMIC) are represented and their voices and perspectives are included in the evaluation (Apgar and Douthwaite 2021; Cornwall and Aghajanian 2017; Green and Mcallister 1998; Guijt 2014). Participatory development of theory of change means that outcomes, pathways and assumptions are defined by the ‘change agents’ in the systems a programme intervenes in, and is assumed to lead to greater ownership over the process and the outcomes (Apgar and Douthwaite 2021). ARISE and TC collaboratively built their theory of change and developed partnership principles with all Hub partners, which then shaped the evaluation designs. GJS held online workshops for partners to create nested TOCs for each of the six organising streams that structure the Hub’s work, ensuring that partners were actively involved in identifying the activities, outputs and outcomes for their workstreams.

In the ARISE case, both the CBPR methodology and equitable partnerships are central to the Hub Theory of Change. CBPR aims to challenge the power relationships that are inherently embedded in western knowledge production (Tremblay et al. 2018). One of the key assumptions is that equitable partnerships can better respond to opportunities and mitigate risks, thus contributing to increased capabilities of people living and working in marginalised contexts. ARISE took a participatory approach to the development of partnership principles. Based on the key assumption and principles, an annual partnership survey is used to review the principles. This allows the Hub to address ongoing challenges and understand different perspectives about what equity means, assess and respond to emerging challenges as a collective. This longitudinal approach helps to assess changes in equitable partnerships over time. Qualitative interviews and discussions with partners help to further understand how equitable partnerships are shaping the research and contribute to building capabilities of urban marginalised people. Participatory discussions (online and in-person) at all levels of the partnership further help to annually review the partnership functioning and principles with all partners involved.

**Fig. 3:**
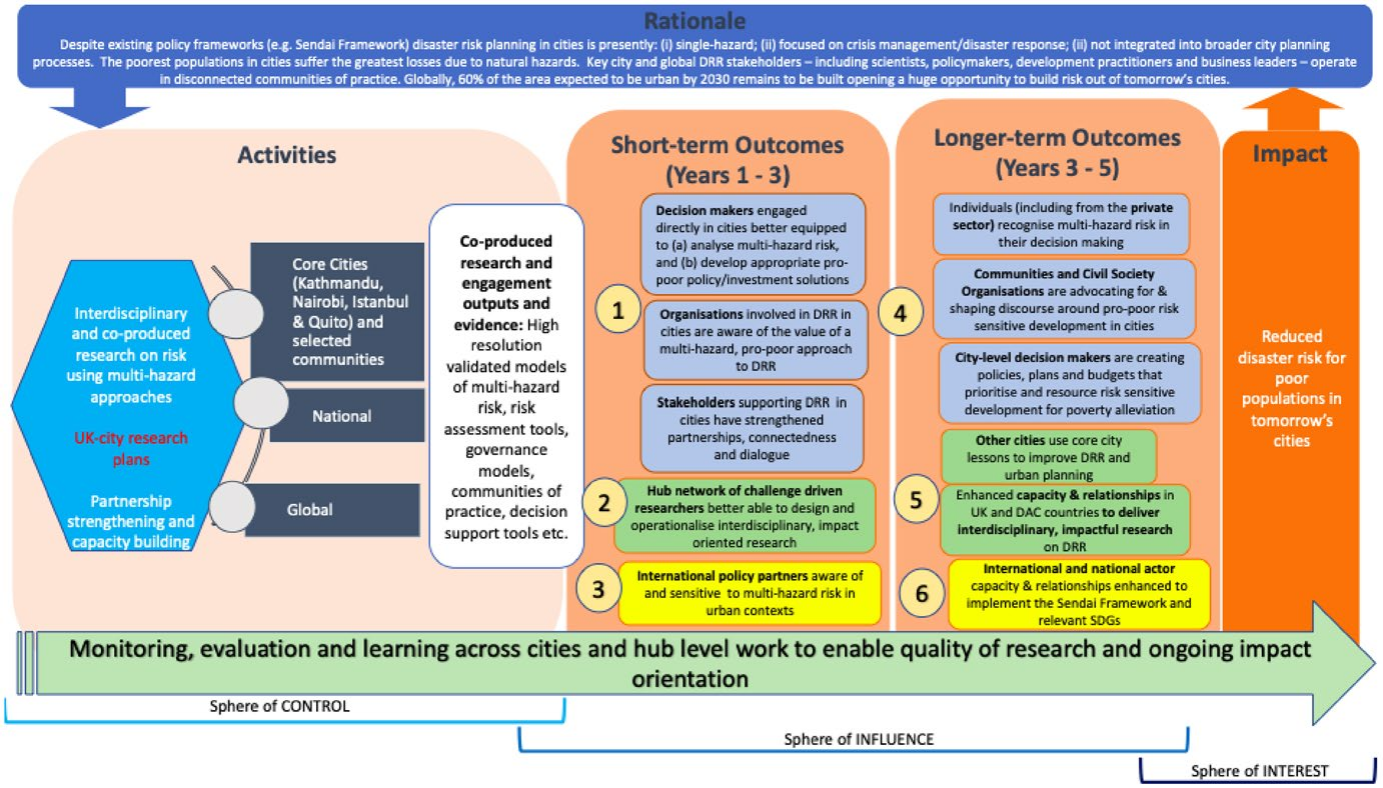
Tomorrow's Cities theory of change

**Fig. 4:**
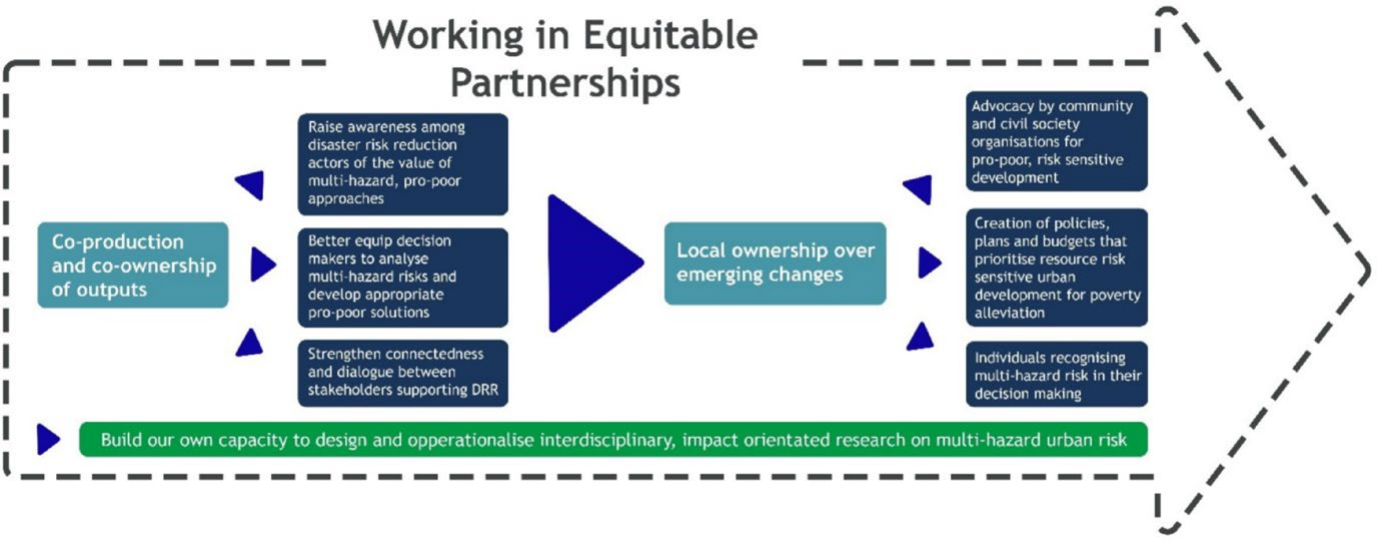
Equitable partnerships theory of change in Tomorrow's Cities (*Source* Price et al 2021)

Contribution analysis was used in the TC case for the overall evaluation approach with participatory development of theory of change and partnership elements (Apgar and Douthwaite 2021; Mayne 2008). The theory of change was constructed with partners during workshops in the inception phase of the Hub (Fig. 3) and three main impact pathways were identified. Figure 4 illustrates how, as an ongoing process (indicated by the large arrow), the Hub theorises that working in equitable partnership is contributing to external outcomes (dark blue boxes) in the disaster risk reduction landscape (through the mechanisms of co-ownership over outputs and research and local ownership of changes) and builds internal capacity to work in ways to support these external outcomes (green box). The subsequent partnership assessment focused on if and how the partnership is working equitably, how this develops over the lifetime of the hub and how it contributes to the outcomes. During annual review and reflection workshops with each of the four country teams (attended by 10 to 36 partners), they identified the core elements of equitable partnership for their country team. In the discussion and synthesis, particular attention was paid to the views of ECRs, LMIC-based and female researchers to ensure that their voices were included in the way partnerships would be evaluated. These core elements were then turned into an evaluative rubric, which was used in the review and reflection workshop with partners to assess country teams’ partnership working. A country-specific survey was generated based on these elements. Social network analysis was completed to assess the connections across the partnership (see Apgar et al. this issue) and in annual reporting all partners were able to provide their perspectives on equity and fairness in the partnership.

## Cross-case Analysis Findings

In this section we look across the five unique cases to compare how the findings of the evaluations were used to address the following power asymmetries: between UK and LMIC partners, academic hierarchies and across gender dynamics. The information in this section comes from the within-case evaluations described in the previous section, the data sources of which included interviews, surveys, reflection sessions, documentation analysis and ethnographic fieldwork.

### Addressing UK-LMIC Power Asymmetries

The evaluation findings in all cases confirmed the existence of power asymmetries early in the life of the programmes between UK and LMIC partners. This is partly structural as funding was required to be disbursed and audited by UK-based institutions, leading to UK-based partners dominating the Hubs at the outset. The pandemic, which interfered with travel, coupled with the unexpected budget cuts frustrated desires to flatten power asymmetries. Remote working made it more difficult to connect with partners across geographic locations, limiting how much partners could learn from each other’s contexts. For example, TC had to cancel their all-Hub conference in May 2020 and GJS could not implement their LMIC-HIC Exchange scheme. Qualitative interviews from ARISE also revealed that the impact of the cuts meant LMIC researchers had limited time to foster South-South partnerships, placing constraints on horizontal learning that were intended to disrupt vertical asymmetries. Despite the focus on equal opportunities and representation from LMICs, the reality across the cases is that all Hubs remain UK-led. For example, all Hubs are based at UK institutions; are led by UK-based PIs; and, in the case of the LD Hub more than 50% of the Hub’s research projects are UK-led.

Within the GJS case, at the local level, the resurgence of the Taliban in Afghanistan and the allied withdrawal in August 2021 revealed a more entrenched and pernicious form of inequity between UK and LMIC partners, which fundamentally challenged the notion of equitable partnerships in the GJS Hub. GJS has an Afghan partner organisation, and from August 2021 has been trying to evacuate its research team, with only limited success. The team are at imminent risk from the Taliban because of their association with UK research funding and the UK’s research priorities in women, peace and security. The risks to the Afghan project partner and the threats they have experienced exponentially outweigh any benefits that accrue to UK-based researchers who rely on them to undertake culturally sensitive fieldwork.^2^ This highlights the additional inequity in risks that different partners are facing when working on UK-based funded R4D.

Across the cases different strategies were adopted to address the UK-LMIC power asymmetries. Hubs were required to undergo restructuring in response to the 2021 budget cuts and used the findings of their partnership evaluations to shape these restructures taking them as an opportunity to increase equity between UK and LMIC partners. Hubs restructured in such a way that ‘workstreams’ or ‘workpackages’ were either led by an LMIC academic (OOH) or jointly led by a HIC and LMIC academic (GJS and TC Hubs). This not only enabled genuine collaboration between partners but also served to model the Hubs’ commitment to partnership at all levels, from project design and delivery through to governance. In the TC Hub the structure of new workstreams were partly informed by case studies of ongoing work produced by LMIC based teams. In other cases the Hubs aimed to address asymmetries by providing new opportunities for all partners: the implementation of a rotating chair of Executive Committee each month to build shared responsibility and opportunities to set agendas and ensure the hub benefited from different chairing styles (ARISE Hub); regular all-Hub open communication forums through Zoom that provided an opportunity for all partners from all countries to connect (TC Hub); mentoring programmes (ARISE) and other opportunities to build LMIC networks (GJS and LD). These processes supported partnerships between LMICs and capacity building at multiple levels, as illustrated by this quote from an ARISE academic:

I’ve developed skills in] leadership and project management so it's been quite useful having their support, I have a somebody I've been paired with in Kenya. Very similar context, we talk we engage. And then at the same time I’m able to support early career researchers. - ARISE Researcher Sierra Leone.

### Addressing Power Asymmetries of Academic Hierarchies

The partnership evaluations in all of the cases identified that there were power asymmetries experienced between senior and junior researchers in the Hubs. These differences also linked to UK-LMIC and gender-based asymmetries, as most senior academics were based in the UK and more likely to be male. Further reflections emphasised that hierarchies are not problematic in and of themselves, and indeed they are necessary features of managing large complex programmes, rather not being explicit about how power works across a hierarchy and paying attention to how opportunities are created or not through hierarchical relationships is problematic.

in a project such as Tomorrow’s Cities there are people who are responsible for other people and for their task, hierarchies in some ways help to manage these responsibilities…the Hub probably does not need to get rid of hierarchies, but rather to discuss them, for example in terms of gender. – TC AAR

Interestingly, the need to work virtually during the pandemic in some cases was reported to increase equity in participation as all researchers were able to attend all meetings regardless of seniority whereas previously, funding limitations, visa inequities or family commitments may have prevented them attending in-person meetings.

Between the cases there were differences in the way that the academic hierarchies impacted on the work of the Hubs. In the ARISE and TC cases because junior staff in LMICs tend to do most of the field and implementation work, their practical fieldwork was most heavily impacted by the pandemic as a direct result of not being able to collect data. In the LD Hub, gaps between ECRs and more senior colleagues were experienced with regards to publication, for example some LD Hub members felt that adequate time was not provided to contribute to publications, while others felt that they did not have sufficient support to lead publications as illustrated in the following quote from LD evaluation:

When ECRs lead a publication, sometimes they fail to recognize the value of including senior delta partners as co-authors. – LD Hub Researcher

Despite mitigation attempts, the impacts of budget cuts were severe and from the evaluations it became clear that in most cases, the impact were felt largely by ECRs (including PhD candidates), both in terms of contracts ending and in terms of their work plans. In the TC and LD cases, more ECRs than senior researchers lost their positions in the Hubs. In the ARISE, TC and LD cases the evaluations found that for those ECRs that were retained, some activities were reduced which impacted on relationships beyond and outside the Hub. Especially in TC and ARISE Hubs it was highlighted by ECRs that the burden of communicating the cuts to the communities, with which they have built relationship, fell on them:

but from the perspective of the communities it's been, it's bringing in issues because they assume they were no cuts. So they assumed that you just decided to cut on activities that were going on... So [they believe] it's either you refuse to continue working with us, or you decided to go and work with other communities and you’re not honest with us. So we've had to spend a lot of time explaining to them, having meetings with them. - ARISE ECR Kenya

In two cases (ARISE and GJS) findings from the partnership evaluations were explicitly used to develop and implement strategies to mitigate against asymmetrical job security which unequally favours tenured or more established staff over ECRs, and gives greater certainty to those with ongoing rather than contracted employment. GJS developed a set of principles for applying the budget cuts equitably across projects and partners and posted those principles on the Hub’s communication platforms for all members to access. The principles ensured that those whose livelihoods depended solely on the Hub were prioritised for Hub funding, while more senior staff in academic or salaried positions reduced or suspended their Hub funding for the year. This approach, while drastically reducing research outputs, maintained the careers of the most vulnerable project partners in civil society and ECRs. This approach to supporting people over projects has meant that the Hub was well-placed to recommence research activity when GCRF funding was restored. ARISE’s strategy was for some senior staff (from HIC and LMICs) to reduce their salaried time to support Hub members on more precarious contracts (usually ECRs). In these two cases, these activities that were grounded in their existing equitable partnerships meant that they could respond to the budget cuts in an equitable manner, guided by co-created values. Being clear on power dynamics and equity from the start, built trust among partners meaning when challenges arise, Hub partners came together in solidarity.

we still are able to work together in all of these difficulties, because of the understanding and the partnership and the kind of relationship we have a lot of trust, we have been able to understand each other, and make sure that whenever there's a challenge, we come together. – ARISE ECR SL

Aside from responses to the budget cuts, in two cases other strategies were implemented to address more problematic power asymmetries between ECRs and more senior colleagues that negatively impact on the work of the Hubs. Hubs established sub-groups that were led by ECRs (TC and LD Hubs). These groups consisted of ECRs from all countries across the Hubs, appropriate disciplines and mixes of genders. The groups had specific purposes, for example modelling, creating household surveys or the development of decision support environments (Galasso et al. 2021). These groups provided ECRs with leadership over specific elements of the Hubs and opportunities to collaborate with colleagues from across different partners. Related to academic publications, the LD Hub opened a dedicated Microsoft Teams channel to attempt to both monitor and encourage equity in the development of publications across the Hub, which resulted in several recent publications that demonstrate co-production between LMIC and UK-based colleagues and are led by ECRs (see Online Appendix).

### Addressing Gender-Based Power Asymmetries

In all but one case, the evaluations found that gender-based power asymmetries were present in the Hub. In the TC Hub the evaluation revealed that in Turkey and Ecuador gender and seniority (with most leaders in the Hub being male) created power asymmetries. In the LD Hub, while female Hub members hold leadership positions, the evaluation showed that decision making processes are dominated by men and the latter more strongly feel they have power to influence decision making. Only the evaluation of the GJS Hub showed that partners strongly believe that their Hub is gender equitable, as gender equity is both a research focus and organising principle of the Hub’s structure and work. The Hub’s statement of ethics, which forms a clause of all partner collaboration agreements, expressly states that no Hub member will be treated less favourably due to gender identity, expression or sexual orientation.

In the four cases with gender-based power asymmetries it was not always directly clear from the evaluations how this impacted on the research of the Hub. Reflections from TC suggested that female members felt that they undertook more ‘invisible work’ during the pandemic inside and outside of the project. Invisible work relates to relationship building, caring responsibilities for staff (e.g. supporting more junior staff with mental health, having informal conversations) or family members. Such activities limited the time they could spend on research activities and consequently led to them missing out on publications. One reason why the impact of gender-based imbalances might not have been directly clear in all cases, is that physical science co-investigators were less likely to see gender as relevant to their work than social scientists. For example, an observation emerging from the OOH case was that researchers who are not often asked to consider the gender dimensions of their research see gender was a ‘political’ exercise:

This dimension simply does not exist in my research. Apart from the "politically correct" character of the request, detailing the reasons for a non-existing “something” is a case of ontological non-sense. - Co-Investigator, One Ocean Hub

In the LD case it was clearer how gender-based power asymmetries influenced their research and outreach activities. The evaluation found that ensuring gender balance in participants in research and outreach activities was a challenge in the Hub, especially those based in LMICs. This difficulty relates to the composition of the in-country research partners and underlying structural practices within the countries in which the LD Hub is working. For instance, at an outreach event in one of the partner countries, all in-country speakers were male (one female senior Co-I who could have engaged was not available). Moreover, the relevant technical government agencies that were invited to participate at the event nominated male speakers who held senior positions within their organisations.

In the GJS case, all of the Hub’s thirty-two *projects* address gender equality directly, and the projects overall represent an innovative and comprehensive engagement with different aspects of gender. The projects’ annual reporting template asks project Co-Investigators to identify the strategies they use for gender inclusion, including how the design of community consultations is either gender-inclusive, or limits gendered participation to ensure the safety and freedom of expression of participants (for example, in some settings women or transgender participants will speak more freely without cis-gender men present).

In response to the evaluation findings of gender-based power asymmetries, activities to mitigate were implemented in the cases, such as: Gender mainstreaming activities through seminars and all-Hub meetings; ensuring equal participation of men and women in activities; and, having a gender champion on the advisory board. The GJS Hub continues to implement its feminist ethics policy, including in its safeguarding policy, which provides avenues for responding to harms that are closely linked to structural inequalities and social power dynamics in which gender plays a role. This safeguarding policy is one of the first of its kind and has been taken up as a model for R4D programmes within the lead partner, London School of Economics.

## Discussion

We took the unique opportunity that our engagement in five large R4D programmes provided to describe approaches and methods to evaluate equitable partnerships and how these evaluations were used to unearth and address power asymmetries within the partnerships. Whereas most research partnership literature focuses on HIC and LMIC power dynamics, we also included power asymmetries based on gender and academic hierarchies. In this final part of the paper, we will first summarise key findings in terms of existing power asymmetries and how they were addressed. The rest of the discussion section will share implications and a framework for future equitable partnership evaluations that expands on previous frameworks (e.g. Fransman et al 2021 and Gomez-Bonnet and Thomas 2015) by centring review and reflection processes alongside a multi-method approach that embraces the multi-layered and complex nature of partnerships in R4D programmes.

### Key Findings on Power Asymmetries and How to Address These

We found that there were intersecting power asymmetries, with more UK-based males in decision making positions in the Hubs. In only one case the evaluation found no gender-based power asymmetries as this Hub prioritises gender equity in their organising principles and topic of research, illustrating that gender-based power asymmetries are not inevitable when prioritised. The evaluations found that academic hierarchies are not inherently problematic, but that it is about being open about how these power dynamics work and providing fair opportunities across the hierarchy. When there is a lack of openness about these dynamics there is a risk of interpersonal power abuses, especially in these large-scale R4D programmes that are often characterised by prioritising production of high quality research over relational aspects of the work. It is hard to capture these tensions both given the sensitivity of this topic and given the learning focused nature of the evaluations presented here that were mainly supported by more junior colleagues, which makes it harder to draw out these issues. The multiple crises that the Hubs were faced with were seen as an opportunity to attempt to increase equity in the Hubs and the evaluation findings were used to inform the restructuring of the Hubs in such a way that LMIC priorities and partners led the work. Especially Hubs who used their equitable partnership principles in responding to the crises identified that this facilitated continuing research work once the crises subsided. However, due to funding cuts it was inevitable that programmes had to readjust some of the work and in most Hubs included in this paper it contributed to a reduction in MEL work and capacity and most of us (the authors) no longer remained involved. Finally, the UKRI funding structure places limits on how much funding can flow to LMIC partners and requires a UK-based lead, putting limits on equity of the partnership from the start. Thus illustrating the important role that funders play in making equitable partnership possible (ESSENCE on Health Research & UKCDR 2022).

### Implications for Evaluation of Equitable Partnerships in R4D: The Case for Adaptive Management and Theory-Based Approaches

Evaluation of equitable R4D partnerships is a nascent field that can benefit from theory-based evaluation to build understanding of how and why working in equitable partnerships works in different contexts (Hoekstra et al. 2020; Price et al. 2021; Schwarz et al. 2021). Our analysis revealed different ways in which equitable partnerships have been included in the theories of change of the five Hubs: as a key activity and as an output that is shaped by capacity and resource building activities that in turn contribute to the Hub’s impact. Equitable partnerships were also part of the critical assumptions that need to be in place for the Hub to be contributing to outcomes. One included case illustrated how a ‘nested’ equitable partnership theory of change was developed that more specifically detailed how working in equitable partnership specifically contributes to the overall programme impacts. Our study showed that developing an in-depth understanding of the central role of equitable partnerships in R4D’s ability to contribute to impact through its inclusion in the theory of change, means that the relational side of R4D is emphasised both in the evaluations and in the doing of R4D. As was illustrated in two cases by how the Hubs responded to the budget cuts by retaining most staff even though that reduced the research productivity of the Hubs.

Participatory approaches to partnership evaluation in the discussed cases meant it focused on what is of importance to the different partners. As illustrated in two cases, the co-developed partnership principles that were used to measure partnership performance against meant that the evaluation reflected the varied cultural backgrounds of partners and thus helped to move away from UK-centric notions of equitable partnerships. In one case this approach meant that there were different definitions and principles of equitable partnerships across the different countries involved in the Hub which allowed to embrace the cultural context of each country and have meaningful definitions of equitable partnerships for all involved. This approach aligns with recent views that engaging with partners’ contexts is essential to addressing power asymmetries (Fransman et al. 2017, 2021). Engagement with the partners’ context can also contribute mutual understanding of why partners may choose to not collaborate or reveal if they have specific needs around collaboration (van Paassen et al. 2022). For example, showing that the Afghani partners in the GJS Hub faced additional safety risks due to their connection with UK funding.

Our cross-case analysis shows the added value of taking a learning-oriented approach to the evaluation of equitable partnerships, rather than simply measuring performance for periodic reporting. The included partnership evaluations were embedded within ongoing R4D programmes and evaluations were used to inform and adapt the ways the Hubs were dealing with power asymmetries and maintaining equitable partnership through crises (covid-19, 2021 UK ODA budget cuts, local crises). Not all cases explicitly described this as part of an adaptive management approach, but the evaluations surfaced that learning was feeding into decision making. Some did this by creating spaces to discuss partnership survey findings with Hub management teams, while others used the intentionally designed learning infrastructures, such as review and reflection sessions. These spaces provided opportunity to deepen the participatory evaluation approach, as well as momentum to push towards use of evaluation findings.

Finally, we need to consider the limitations to fostering and evaluation ‘equitable’ partnerships given some of our findings speak to deeply entrenched power asymmetries, including those resulting from colonial histories, funding streams, knowledge hierarchies and paternalism. Some have argued that building equitable partnerships within this context is impossible, because the colonial model of GCRF “maintains paternalistic and colonial assumptions around Northern researchers solving problems located in the South and building Southern research capacity” (Nwako et al. 2023, p. 14).

### A Framework of Equitable Partnership Principles and Evaluation Measures

**Fig. 5:**
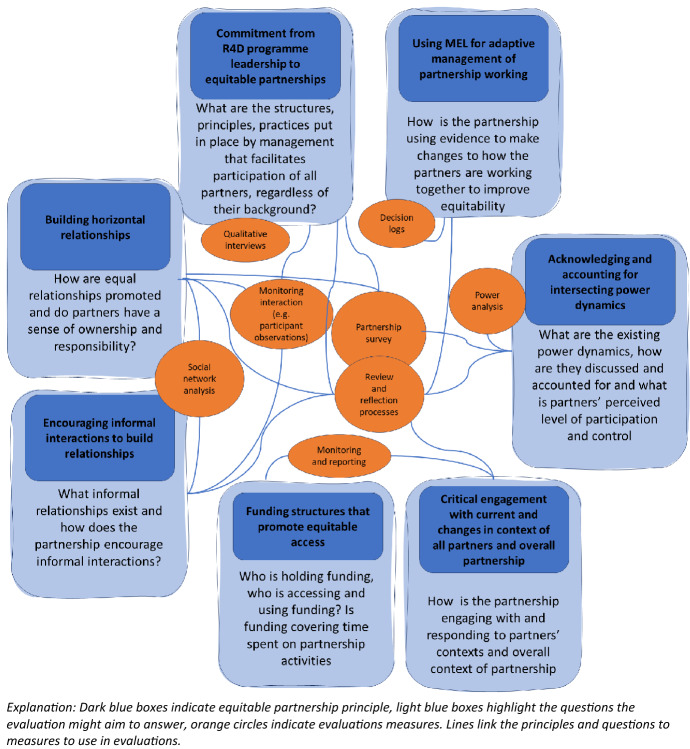
Equitable partnership principles and evaluation measures framework

We finish this paper with a framework (Fig. 5) for evaluating equitable partnership based on principles from the literature (Dodson 2017; Fransman et al. 2017; Hoekstra et al. 2020; Larkan et al. 2016; Price et al. 2021; Snijder et al. 2020) to which we add the need to acknowledge and address power asymmetries and using MEL for adaptive management of partnership working. This evaluation framework embraces the complexity of R4D partnerships by including operational (funding structures, leadership and MEL systems), relational (informal and horizontal relationships), contextual (engagement with partners’ contexts) and power-related elements of equitable partnership working. This framework encourages evaluations of equitable partnerships to go beyond the use of just partnership surveys, rather use multiple methods and especially centre review and reflection processes with all partners to embrace a learning-oriented approach in which the findings from the evaluation are actively used to inform adaptations to the partnership.

The *support of programme leadership* for joint ownership and equitable ways of working is essential for equitable partnerships. In the included cases, the leadership teams used evaluation and learning data to identify issues as they emerged and adapted approaches accordingly. The evaluations in this paper assessed this using interviews with management, surveys with programme staff, reflective practices (e.g. review and learning workshops) and by monitoring interactions from leadership with the rest of the programme.

*Establishing horizontal relationships* in which everyone has a sense of ownership and responsibility and is respected for the expertise they contribute to the partnership was identified in our evaluations and others (Hoekstra et al. 2020; Price et al. 2021) as essential to build equitable partnerships. Relationships between partners can be assessed using social network analysis (see Apgar et al. this issue). Surveys with partners in this and other studies have been used to measure how much ownership and sense of responsibility different partners experience (Hardy et al. 2003; Pasanen 2016; Wagemakers et al. 2010). Review and reflection processes can help partners reflect on their relationships. Closely linked to horizontal relationships is the increasing acknowledgement in the partnership literature that successful partnerships are built on *close interpersonal relationships and friendships* (Fransman et al. 2021; Price et al. 2021). Mapping interactions between partners through social network analysis and monitoring of meetings can help bring to life how partnerships are being shaped by informal relationships.

Partnerships and their evaluations need to *critically engage with the context* within which the partnership is operating in order to address power asymmetries (Fransman et al. 2017, 2021). The context of the partners shapes their contribution and accounting for this can ensure unique contributions are acknowledged. Included cases in this study illustrated how partnership evaluations were explicitly co-designed with all partners to ensure the definitions and understanding of equitable partnerships reflected different cultural understandings, which were then incorporated into partnership surveys. Review and reflection processes and monitoring and reporting of interactions of the partnership with the wider context can be used in partnership evaluations to better understand how the partnership is interacting with contextual factors that influence it.

An important contextual factor that shapes equitability of R4D partnerships is the *funding landscape*. Our experience suggest that the funding arrangements dictated by UKRI created a structural barrier to equity, in particular, the restrictions on how much and in what ways partners could be funded. A focus on equitable partnerships helped some Hubs implement structures that promote equitable access to discretionary funding held by the Hubs to overcome this barrier. Exploring questions on who is holding funding and accountability and who can access funding are important to explore in equitable partnerships. Monitoring funding flows within the R4D programme can provide valuable insights. Furthermore, if equitable partnerships are prioritised, then relational work undertaken within R4D programmes should be funded as part of the core work, so that it is not perceived as extra work partners are required to do in addition to their research, but that it is a fundamental element of the research.

Additional contextual factors such as partners’ academic, cultural and gender backgrounds shape *intersecting power dynamics in R4D partnerships* (e.g. gender relations and seniority are influenced by cultural contexts). Partnership surveys were used to measure partners’ perceived level of participation. Review and reflection processes as part of MEL systems, helped partners to assess collectively how power is being used, discussed, and mitigated for. While not used in any of our cases, evaluations can use power analysis to identify power balances at the start and how they change over time. *Surfacing learning for adaptive programme management*, mentioned previously, can facilitate partners learning about and adapting to their contexts. Alongside the already mentioned review and reflection processes, decision logs can help to track adaptative decisions and provide evidence of learning in use.

### Strength and Limitations of this Research

The multi-case study approach we took means that each Hub conceptualised and evaluated equity in ways that was appropriate for their specific context. While this may be considered a weakness in terms of lacking a systematic approach to comparison, it is also a strength as we demonstrated a variety of approaches and distil learning from different practical ways of working. The differences between the Hubs in terms of scope and shape (see Table 1) make it difficult to compare partnership performance across them. Nonetheless, we contend that there are enough similarities (they are all inter-disciplinary, were funded within the same funding programme and experienced similar challenges of both COVID-19 and unexpected large aid cuts) to justify the design. There are twelve Hubs in the overall GCRF cohort and most of them were involved in initial conversations across MEL teams within the Hub, yet not all Hubs participated in this joint analytical and synthesis process. We assume that the process led to self-selection of the five Hubs that prioritised equitable partnerships and may have led to missing out on interesting learning on partnerships in Hubs where they are less prioritised. A smaller number of cases allowed us to provide some in-depth contextual information, practical examples and nuance which we feel is necessary.

This paper did not specifically look at the inclusion of the ‘beneficiaries’ for whom the projects aimed to improve outcomes. As other large R4D partnerships, the Hubs represent partnerships between academic institutions, NGOs, government and civil society organisations. Further research could explore whether and how the most marginalised can be directly involved in these partnerships so benefits might more directly trickle down to them (Dekker and Pouw 2022).

## Conclusion

Tackling complex development challenges requires R4D programmes to work in partnership with academic and non-academic partners from HIC and LMIC and diverse disciplinary backgrounds. For such partnerships to thrive and optimally integrate all partners’ perspectives and expertise, they need to be working in equitable ways. Our cross-case analysis showed that performance monitoring and theory-based evaluation were used in five large R4D programmes to help them learn about their partnership functioning and adapt to address power asymmetries to increase the equitability of their partnerships. Theory-based evaluation and adaptive management approaches that combine an array of tools are ideally placed to support R4D partnerships to address power asymmetries.

## Supplementary Information

Below is the link to the electronic supplementary material.

Supplementary file1 (DOCX 19 KB)

## Data Availability

All research materials can be accessed through the relevant institutional data management systems that are UKRI compliant. All hubs had extensive data management plans that had to be approved by UKRI.

## Footnotes

1 The Flexible Funding was funding that each Hub already had committed from UKRI, but that did not been assigned to specific research activities yet, with the idea that this would be flexible to use for project ideas that emerged from the first phase of the Hubs.

2 This risk exacerbates the already entrenched asymmetries between HIC researchers and LMIC project partners which the Hub has worked very hard to mitigate through its governance, policies and partnerships strategy.
