# Supplementary material for: How are Research for Development Programmes Implementing and Evaluating Equitable Partnerships to Address Power Asymmetries?
Source: Eur J Dev Res. 2023 Feb 23;35(2):351–79. doi: 10.1057/s41287-023-00578-w (PMC9947878; doi:10.1057/s41287-023-00578-w)
Supplement: Supplementary file 1 — Supplementary file1 (DOCX 19 KB) [file 41287_2023_578_MOESM1_ESM.docx]

**Appendix: ECR-led publications from Living Deltas Hub**

Kumar, A., Tuladhar, N., & Pal, I. (2022). Demystifying Impacts of Cyclone Amphan 2019 Amid COVID-19 Pandemic in West Bengal, India. In S. Kolathayar, I. Pal, S. C. Chian, & A. Mondal (Eds.), *Civil Engineering for Disaster Risk Reduction* (pp. 461–478). Springer. https://doi.org/10.1007/978-981-16-5312-4_30

Moorhouse, H. L., Roberts, L. R., McGowan, S., Panizzo, V. N., Barker, P., Salehin, M., Do, T. N., Nguyen Thanh, P., Rahman, M. F., Ghosh, T., Das, S., Hackney, C., Salgado, J., Roy, M., Opel, A., Henderson, A. C. G., & Large, A. R. G. (2021). Tropical Asian mega-delta ponds: Important and threatened socio-ecological systems. *Geo: Geography and Environment*, *8*(2), e00103. https://doi.org/10.1002/geo2.103

Pramanik, M., Szabo, S., Pal, I., Udmale, P., O’Connor, J., Sanyal, M., Roy, S., & Sebesvari, Z. (2021). Twin Disasters: Tracking COVID-19 and Cyclone Amphan’s Impacts on SDGs in the Indian Sundarbans. *Environment: Science and Policy for Sustainable Development*, *63*(4), 20–30. https://doi.org/10.1080/00139157.2021.1924575

Pramanik, M., Szabo, S., Pal, I., Udmale, P., Pongsiri, M., & Chilton, S. (2022). Population health risks in multi-hazard environments: Action needed in the Cyclone Amphan and COVID-19 – hit Sundarbans region, India. *Climate and Development*, *14*(2), 99–104. https://doi.org/10.1080/17565529.2021.1889948

Rahman, K. F., Pal, I., Szabo, S., Pramanik, M., & Udmale, P. (2021). Chapter 3 - Transboundary water risk governance frameworks in deltaic socio-economic regions: A case study of river deltas in Bangladesh, India, and Vietnam. In I. Pal, R. Shaw, R. Djalante, & S. Shrestha (Eds.), *Disaster Resilience and Sustainability* (pp. 49–72). Elsevier. https://doi.org/10.1016/B978-0-323-85195-4.00011-1

Udmale, P., Pal, I., Szabo, S., & Pramanik, M. (2021). 2021-IEJ SpecialIssue1AJan2021 BangabandhuChair Volume2-112-123. *International Energy Journal*.
